# Frequent germplasm exchanges drive the high genetic diversity of Chinese-cultivated common apricot germplasm

**DOI:** 10.1038/s41438-021-00650-8

**Published:** 2021-10-01

**Authors:** Qiuping Zhang, Diyang Zhang, Kang Yu, Jingjing Ji, Ning Liu, Yuping Zhang, Ming Xu, Yu-Jun Zhang, Xiaoxue Ma, Shuo Liu, Wei-Hong Sun, Xia Yu, Wenqi Hu, Si-Ren Lan, Zhong-Jian Liu, Weisheng Liu

**Affiliations:** 1 Liaoning Institute of Pomology, Yingkou, 115009 China; 2 Key Laboratory of National Forestry and Grassland Administration for Orchid Conservation and Utilization at College of Landscape Architecture, Fujian Agriculture and Forestry University, Fuzhou, 350002 China; 3 BGI Institute of Applied Agriculture, BGI-Agro, Shenzhen, 518210 China; 4 Institute of Vegetable and Flowers, Shandong Academy of Agricultural Sciences, Jinan, 250100 China; 5 Zhejiang Institute of Subtropical Crops, Zhejiang Academy of Agricultural Sciences, Wenzhou, 325005 China

**Keywords:** Plant molecular biology, Population genetics, Genome

## Abstract

The genetic diversity of germplasm is critical for exploring genetic and phenotypic resources and has important implications for crop-breeding sustainability and improvement. However, little is known about the factors that shape and maintain genetic diversity. Here, we assembled a high-quality chromosome-level reference of the Chinese common apricot ‘Yinxiangbai’, and we resequenced 180 apricot accessions that cover four major ecogeographical groups in China and other accessions from occidental countries. We concluded that Chinese-cultivated common apricot germplasms possessed much higher genetic diversity than those cultivated in Western countries. We also detected seven migration events among different apricot groups, where 27% of the genome was identified as being introgressed. Remarkably, we demonstrated that these introgressed regions drove the current high level of germplasm diversity in Chinese-cultivated common apricots by introducing different genes related to distinct phenotypes from different cultivated groups. Our results highlight the consideration that introgressed regions may provide an important reservoir of genetic resources that can be used to sustain modern breeding programs.

## Introduction

Crop germplasms, particularly those from the centers of origin, provide critical resources for exploring and conserving genetic and phenotypic diversity for breeding applications^1,2^. The diversity of crop germplasm has been suggested to have important implications for breeding sustainability and crop improvement, as it determines the sustained ability of plant breeders to develop new high-quality varieties^3^. Hence, it is essential to characterize the factors driving and maintaining germplasm diversity in crops, a consideration that has largely been ignored in previous plant-breeding research.

The common apricot (*Prunus armeniaca* L.) belongs to the subgenus *Prunophora* of the genus *Prunus* in the Rosaceae family and has been widely grown in temperate zones, primarily in its cultivated form. Documented evidence suggests that common apricots originate from China and Central Asia and are dispersed outward^4–6^. The common apricot germplasm in China has been speculated to be the oldest, most diversified, and the most currently underexplored resource^7^. Recorded in ancient Chinese literature, the first apricot-cultivation event occurred approximately 3000–4000 years ago^8^, and this represents the earliest apricot domestication in the world. The genetic structure of Chinese apricots that was revealed by molecular markers supported the existence of five major ecological groups, including those from North China (NC), Northwest China (NWC), Northeast China (NEC), Southeast China (SEC), and Xinjiang (XJ), with frequent germplasm exchanges occurring among these groups^9^. A long history of cultivation in combination with varied ecological groups and frequent germplasm-migrant events enables Chinese apricot to serve as an attractive model to investigate the factors responsible for germplasm diversity in crops.

Here, we report a chromosomal-level genome assembly of *P. armeniaca* “Yinxiangbai”, and we resequenced the whole genomes of 150 apricot accessions from China that covered four of the five major ecological groups and 30 occidental accessions. In this study, we addressed the population genomics of apricots with an emphasis on germplasm-exchange events, and we further elucidated the role of this process in shaping the germplasm diversity of the common apricot in China.

## Results

### Genome assembly and annotation

*Prunus armeniaca* (“Yinxiangbai”), a native diploid cultivar from Lintong, Shanxi Province, North China, was selected for whole-genome sequencing. We generated a total of 45.73 Gb of raw data with a 350-bp insert-size library and 47.52 Gb (PacBio) and 82.37 Gb (Nanopore) of long reads (Supplementary Table 1). A 17-mer analysis revealed that *P. armeniaca* “Yinxiangbai” possessed a genome size of 264.4 Mb with a heterozygosity rate of 0.99% (Supplementary Fig. 1). The integration of the short and long reads yielded a final genome size of 251.19 Mb and a contig N50 of 4.04 Mb (Supplementary Table 2), both parameters were much larger than those from previously published reports (221.9 Mb with a contig N50 = 1.02 Mb)^10^. The assembled contigs were further anchored to eight linkage groups using linkage maps (Supplementary Fig. 2). Further application of the Hi-C data yielded a total length of 251.19 Mb (Supplementary Table 3), a scaffold N50 of 30.98 Mb, and a contig-anchoring rate of 97.04%, thus representing the highest-quality reference genome ever reported for the *Prunus* genus (Supplementary Fig. 3; Supplementary Table 3). The BUSCO (Benchmarking Universal Single-Copy Orthologs) assessment^11^ revealed that the assembled genome could represent up to 96.20% of the complete *P. armeniaca* “Yinxiangbai” genome (Supplementary Table 4).

Gene model prediction identified 29,230 protein-coding genes (Supplementary Table 5), and this was comparable to that of other *Prunus* species within the Rosaceae family. Of these genes, 91.49% could be functionally annotated in at least one of the five databases (Supplementary Table 6). A total of 46.78% of the “Yinxiangbai” genome was identified as repetitive sequences (Supplementary Table 7), which was much higher than that in *P. armeniaca* “Chuanzhihong” (38.28%)^10^ and other sequenced *Prunus* species (mume [45%], sweet cherry [43.8%], and peach [29.6%])^12–15^.

### Resequencing and variant calling in apricot groups

A total of 180 apricot accessions spanning the geographic ranges of the major ecological groups were selected for whole-genome resequencing (Supplementary Table 8). Specifically, these resequenced individuals included 23 wild apricots, seven from Xinjiang (XJ_W), six from Northwest China (NWC_W), seven *P. sibirica* L., and three *P. mandshurica* (PsPma, wild relatives of common apricot) from Koehne, and 136 cultivated common apricots from Xinjiang (XJ_C, *N* = 29), Northwest China (NWC_C, *N* = 28), North China (NC_C, *N* = 38), Northeast China (NEC_C, *N* = 11), and other occidental countries (West_C, *N* = 30 from the Mediterranean, Eastern and Western Europe, North America, and Australia). Additionally, 21 cultivated kernel-using apricots (KU_C, domesticated from common apricot)^16^ were also resequenced.

A total of 1.09 Tb of sequencing data possessing an average sequencing depth of 21.42 × per individual was generated (Supplementary Table 9). Variation calling using GATK yielded a final set of 10,155,091 SNPs with an average density of 40 SNPs per kb (Supplementary Table 10).

### Genetic diversity and differentiation among apricot groups

Nucleotide diversity (*π*) and gene diversity/heterozygosity (*H*_*E*_) were both calculated across the genome to estimate the genetic diversity of the entire apricot group and of different apricot groups. *π* and *H*_*E*_ for the whole apricot population were estimated to be 6.18E-3 and 6.16E-3, respectively, and either parameter was much higher than those of peach^17^ and pear^18^. The wild apricot relatives (PsPma) exhibited the highest level of genetic diversity (*π* = 6.53E-3, *H*_*E*_ = 6.21E-3), followed by NEC_C, NWC_W, KU_C, XJ_W, and West_C, while the cultivated group XJ_C possessed the lowest level (*π* = 5.19E-3, *H*_*E*_ = 5.10E-3) (Table 1).

**Table 1:** Genetic diversity and Tajima’s D in apricots

Group	*N*	*π* (10^−3^)	*θ*_W_ (10^−3^)	*H*_*E*_ (10^−3^)	Tajima’s D
A: NWC_C:	28	5.48	5.6	5.38	−0.0449
B: NC_C	38	5.24	5.39	5.17	−0.0841
E: West_C	30	5.26	4.6	5.17	0.5485
X: XJ_C	29	5.19	4.48	5.10	0.5975
Yb: NWC_W	6	6.14	6.40	5.63	−0.1443
Ya: XJ_W	7	5.68	5.56	5.28	0.1514
R: KU_C	21	5.74	5.62	5.6	0.1035
H: NEC_C	11	6.21	6.22	5.93	0.0375
Y: PsPma	10	6.53	6.96	6.21	−0.2012
Excluding E, Y	140	6.07	6.74	6.05	−0.3083
Excluding E, Y, R	119	5.94	6.54	5.91	−0.2900
Including A, B, X, H	106	5.88	6.43	5.85	−0.2745
Whole population	180	6.18	6.95	6.16	−0.3441

Note: *N* denotes the sample size. Diversity is described according to nucleotide diversity (*π*), Watterson’s estimator (*θ*_W_), and gene diversity/heterozygosity (*H*_*E*_) and is reported per bp. The cultivated accessions included accessions from NWC_C (Northwest China), NC_C (North China), West_C (including accessions from the Mediterranean, Eastern and Western Europe, North America, and Australia), NEC_C (Northeast China), XJ_C (Xinjiang China), and KU_C (kernel-using apricot accessions); wild apricots included XJ_W (Xinjiang) and NWC_W (Northwest China), and the *P. armeniaca* close relatives (*P. sibirica* and *P. mandshurica*)

We next calculated Wright’s fixation index (*F*_*ST*_) to estimate the genetic differentiation among these apricot groups. First, we assessed the differentiation level between the wild apricot relatives (PsPma) and other groups, as this may provide some insights into the breeding history of different cultivars. Our data demonstrated that XJ_C possessed the highest differentiation level from PsPma, followed by West_C, NC_C, NWC_C, XJ_W, KU_C, NWC_W, and finally NEC_C (Supplementary Table 11). We then examined the differentiation level between the wild and cultivated groups. The lowest differentiation level from NWC_W was found to be for NEC_C, followed by NWC_C, NC_C, KU_C, and West_C. The highest value was for XJ_C. The lowest differentiation level from XJ_W was determined to be for NEC_C, followed by XJ_C, NWC_C, West_C, KU_C, and NC_C. We were also interested in determining the differentiation levels between XJ_C and other cultivated groups. Our results revealed that NWC_C exhibited the lowest differentiation level, followed by West_C, NC_C, KU_C, and NEC_C (Supplementary Table 11).

### Population structure of apricots

**Fig. 1:**
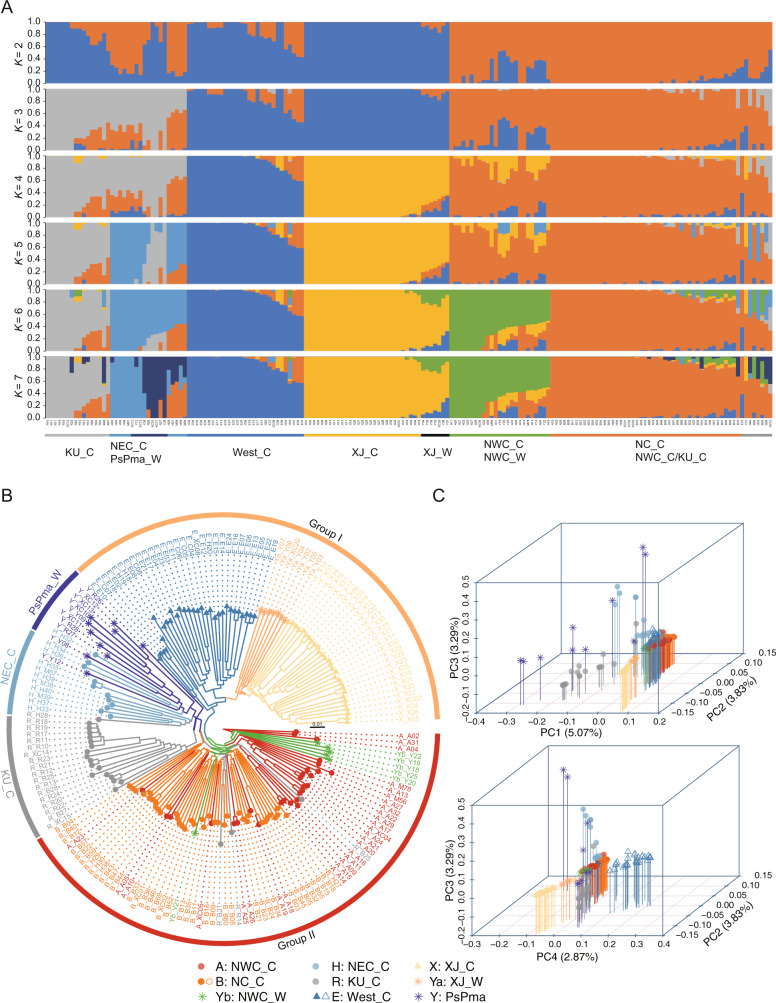
The population structure that was inferred based on genetic variations. **A** The population structure of 180 apricot accessions, **B** an unrooted phylogenetic tree, and **C** principal component analysis (PCA)

We inferred the population structure using the program ADMIXTURE and predefined the number of genetic clusters *K* from 2 to 7. As the cross-validation (CV) value was the lowest for *K* = 6 (Supplementary Fig. 4), we focused on the results for *K* = 6. The results yielded six distinct population clusters that included Cluster 1 (16 accessions highlighted in gray, primarily KU_C), Cluster 2 (19 accessions highlighted in light blue, primarily NEC_C/PsPma), Cluster 3 (29 accessions highlighted in dark blue, primarily West_C), Cluster 4 (36 accessions highlighted in yellow, primarily XJ_C and XJ_W), Cluster 5 (25 accessions highlighted in green, primarily NWC_C and NWC_W), and Cluster 6 (55 accessions highlighted in orange, primarily NC_C) (Fig. 1A). Notably, each cluster included individuals with admixed ancestry, thus implying potential introgression. For example, in Cluster 1, 10 of the 16 individuals that possessed a background from Cluster 6; 11 of 19 individuals in Cluster 2 possessed backgrounds from Cluster 1, Cluster 6, or both; and NWC_W accessions in Cluster 5 and 14 accessions in Cluster 6 possessed the most complex backgrounds exhibiting a mixture of ancestries from at least three other clusters. The constructed unrooted neighbor joining tree and the principal component analysis (PCA) of the 180 accessions revealed a similar pattern, and genetic discrimination was observed for six clusters (Fig. 1B, C).

### Introgression among apricot groups

**Fig. 2:**
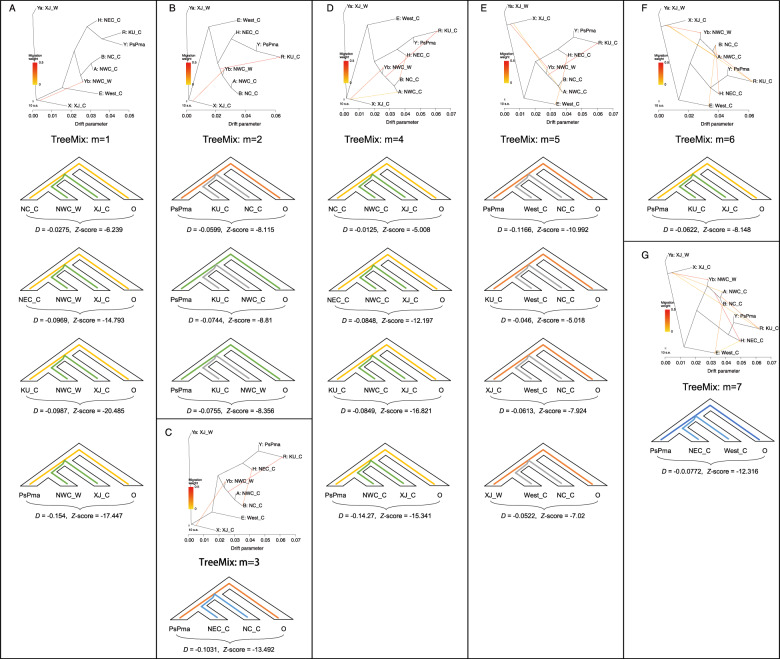
Gene-flow patterns that were detected among apricot groups using TreeMix and *D* statistics. **A** through **G** correspond to migration events from *m* = 1 to 7. *D* statistics for different quadruples of apricot populations (P1, P2, and P3 outgroup) were applied to verify the migration event detected in TreeMix. Negative *D* values indicate that P2 shares more derived alleles with P3 than does P1. P2 and P3 were fixed by two groups to test the gene-flow patterns between them, and P4 was fixed to peach

TreeMix analysis based on log-likelihood and residual variance values suggested the presence of seven migration events (Fig. 2; Supplementary Table 12), and the first event was determined to have occurred from XJ_C to NWC_W (*m* = 1), with the introgressed region covering 8.66% of the genome (Supplementary Table 13). This event led to the adaptive introgression of 2957 genes that were predominantly enriched in pathways related to carbohydrate metabolism (level 2 in the KEGG pathway) (e.g., pyruvate metabolism, amino sugar and nucleotide sugar metabolism, and O-glycan biosynthesis [subpathways in the pathway of level 2]), lipid metabolism (fatty acid degradation, cutin, suberine and wax biosynthesis, and linoleic acid metabolism), and amino acid metabolism (phosphonate and phosphinate metabolism, lysine metabolism, and tyrosine metabolism) (Supplementary Tables 14 and 15).

The second migration event was directed from the ancestor of three groups (NWC_W, NWC_C, and NC_C) to KU_C (*m* = 2), with the introgressed region covering 7.72% of the genome and involving a total of 2,340 introgressed genes (Fig. 2; Supplementary Table 13). Functional enrichment analysis revealed that these genes were significantly enriched in lipid metabolism (ether lipid metabolism, fatty acid biosynthesis and degradation, and cutin, suberine, and wax biosynthesis), metabolism of other amino acids (phosphonate and phosphinate metabolism, glutathione metabolism, and beta-alanine metabolism), amino acid metabolism (lysine biosynthesis and degradation, valine, leukine and isoleukine degradation, and tryptophan metabolism), terpenoid and polyketide metabolism (diterpenoid biosynthesis), carbohydrate metabolism (fructose and mannose metabolism, amino sugar and nucleotide sugar metabolism, propanoate metabolism, pyruvate metabolism, and glycolysis/gluconeogenesis), and the biosynthesis of other secondary metabolites (flavonoid biosynthesis and stilbenoid, diarylheptanoid, and gingerol biosynthesis) (Supplementary Tables 14 and 15).

The third event occurred from NC_C to NEC_C (*m* = 3) and resulted in 10.77 Mb of introgressed regions, covering 4.28% of the genome (Fig. 2; Supplementary Table 13). These regions contained 1404 introgressed genes. Significantly enriched pathways of these genes included replication and repair (base-excision repair, homologous recombination, and nucleotide-excision repair), biosynthesis of other secondary metabolites (flavonoid biosynthesis, flavone and flavonol biosynthesis), and metabolism of other amino acids (glutathione metabolism, taurine, and hypotaurine metabolism) (Supplementary Tables 14 and 15).

The fourth event was from XJ_C to NWC_C, where 21.94 Mb (8.73%) of the genome was predicted to be introgressed, including 2,889 introgressed genes (Fig. 2; Supplementary Table 13). These genes were primarily involved in lipid metabolism (linoleic acid metabolism, glycerophospholipid metabolism, and steroid biosynthesis), translation (RNA transport, mRNA-surveillance pathway, and ribosome biogenesis), and replication and repair (base excision repair, mismatch repair, nucleotide-excision repair, DNA replication, and nonhomologous end joining) (Supplementary Tables 14 and 15).

**Fig. 3:**
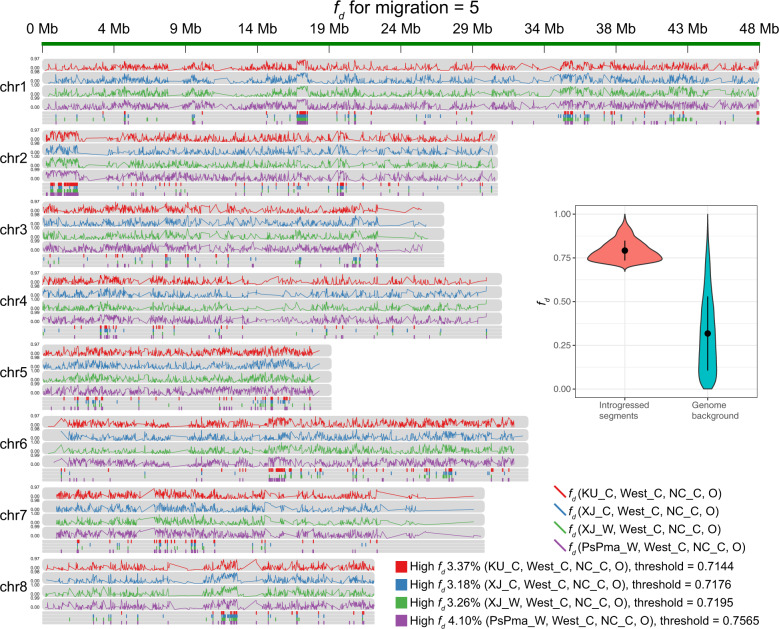
Genomic introgression regions identified among apricot populations. For each chromosome, the four upper panels present the distribution of *f*_*d*_ values for four taxon (X WestC, NC_C, and peach) along each chromosome (X represents KU_C, XJ_C, XJ_W, and PsPma), while the four lower panels highlight the candidate introgressed regions with the top 5% *f*_*d*_ values in different colors (X, XJ_C, XJ_W, and PsPma). The violin plot presents the distribution *f*_*d*_ values of the introgressed regions and genome background for introgression between the apricot populations West_C and NC_C for X setting to XJ_C

The fifth event was observed from NC_C to West_C, where 7.15% (17.97 Mb) of the genome, including 2354 genes, was identified as being introgressed (Figs. 2, 3; Supplementary Table 13). These genes were primarily involved in the metabolism of terpenoids and polyketides (zeatin biosynthesis), amino acid metabolism (lysine, glutathione, tryptophan, tyrosine, taurine, and hypotaurine), glycan biosynthesis and metabolism (glycosaminoglycan degradation and N-glycan biosynthesis), lipid metabolism (sphingolipid metabolism, ether lipid metabolism, and arachidonic acid metabolism), and carbohydrate metabolism (pentose phosphate pathway) (Supplementary Tables 14 and 15).

The sixth event was identified from XJ_C to KU_C and included a 10.25-Mb introgressed region (1245 genes) (Fig. 2). Significantly enriched pathways included amino acid metabolism (valine, leukine, and isoleukine biosynthesis), energy metabolism (carbon fixation), lipid metabolism (glycerophospholipid metabolism and ether lipid metabolism), metabolism of other amino acids (phosphonate and phosphinate metabolism), metabolism of terpenoids and polyketides (diterpenoid biosynthesis), glycan biosynthesis and metabolism (glycosylphosphatidylinositol [GPI]-anchor biosynthesis), and metabolism of cofactors and vitamins (pantothenate and CoA biosynthesis) (Supplementary Tables 14 and 15).

The last event was predicted to occur from West_C to NEC_C (Fig. 2), and 3.77% of the genome (including 1,165 genes) was identified as introgressed (Supplementary Table 13). Pathways related to carbohydrate metabolism (propanoate metabolism), signal transduction (plant hormone signal transduction), amino acid metabolism (valine, leukine and isoleukine degradation and lysine biosynthesis), and metabolism of cofactors and vitamins (riboflavin metabolism) were significantly enriched (Supplementary Tables 14 and 15).

## Discussion

### Genetic diversity and structure of Chinese common apricot germplasm

Although the genetic diversity and structure of Chinese common apricot germplasm has been characterized elsewhere using SSR markers^19,20^, the current knowledge is far from complete. This is primarily due to the limited information available regarding microsatellite markers. To overcome this limitation, we sequenced the entire genome of “Yinxiangbai” and resequenced a total of 180 apricot accessions to comprehensively analyze the genetic diversity and structure of the Chinese common apricot.

Overall, the entire group of Chinese-cultivated common apricots (without PsPma, KU_C, XJ_W, and NWC_W) exhibited much higher genetic diversity than did West_C (including accessions from the Mediterranean, Eastern and Western Europe, North America, and Australia), suggesting that Chinese apricots possessed the most diversified germplasms. This result was consistent with those of previous studies based on SSR markers that reported that the total number of alleles was the highest in Chinese apricots^4,20–22^.

We next investigated the diversity of different Chinese-cultivated groups, and we observed that the diversity exhibited a gradual increase from west to east, where the lowest level recorded was in XJ_C and the highest level was in NEC_C. Notably, each group, with the exception of XJ_C, possessed much higher genetic diversity than West_C, and the diversity of NC_C and NEC_C was even higher than that of the wild accessions from Xinjiang (XJ_W), which has been regarded as the origin of *P. armeniaca*. Given that the common apricot originated in Xinjiang (western China) and spread eastward in China, the observed gradual increase in diversity was striking, as it is generally believed that genetic diversity should decrease from the center of origin outward^23^. Our results have two important implications. Specifically, (1) the germplasm from the center of origin (e.g., Xinjiang) alone cannot represent the whole germplasm of Chinese-cultivated common apricot, and (2) there should be an evolutionary driver that enhances the genetic diversity.

The observed variation in genetic diversity hinted at differentiation between groups. Indeed, population-structure analysis revealed that the four studied Chinese-cultivated common apricot groups could be classified into four genetic clusters, which was consistent with previous reports^8^ (reviewed by Zhang and Liu, 2018). The differentiation level with the accessions from the center of origin (XJ_W), as measured by *F*_*ST*_, also exhibited a gradual increase from west to east.

### Evidence of introgression that contributes to high diversity in Chinese common apricot germplasm

Although a previous study^9^ has suggested that frequent germplasm exchanges occur among different Chinese apricot groups, the study provided no details regarding to what extent and how germplasm exchanges contributed to the observed high diversity of Chinese apricot germplasm.

Based on the admixture analysis of the whole genomes for 180 accessions, we identified seven migration events among the different groups, and a total of approximately 27.73% of the genome was identified as candidate-introgressed regions (Supplementary Table 13). To determine whether these introgression events contributed to the observed high germplasm diversity of the whole Chinese common apricot group, we examined the genetic diversity before and after removing these introgressed regions. As anticipated, removing these introgressed regions led to an ~8.6% reduction in genetic diversity (*π* = 6.18E-3 before *vs*. 5.64E-3 after; *H*_*E*_ = 6.16E-3 before *vs*. 5.63E-3 after). Strikingly, the reduced genetic diversity was similar to that of West_C (π = 5.64E-3 vs. 5.26E-3; *H*_*E*_ = 5.63E-3 vs. 5.17E-3).

Next, we investigated how these migration events contributed to the high germplasm diversity. To achieve this, we conducted functional enrichment of the introgressed genes in each event. Interestingly, we determined that most events were enriched in the same pathways (KEGG level 2) but differed in subpathways. For example, although introgressed genes were observed for the migration events from XJ_C to NWC_W (Fig. 2B), from the ancestor of NWC_W, NWC_C, and NC_C to KU_C (Fig. 2B) and from NC_C to West_C (Fig. 2E), they were significantly enriched in the carbohydrate-metabolism pathway (Supplementary Table 15). The former event was primarily related to the subpathways of fructose and mannose metabolism, amino sugar and nucleotide sugar metabolism, propanoate metabolism, pyruvate metabolism, and glycolysis/gluconeogenesis, whereas the latter event was primarily related to the subpathway of the pentose phosphate pathway. In the former, fructose and mannose were the major sugar components in apricot fruits^24^, and in the latter, pentose was one of the sugar components. These differences could lead to distinct favored traits (e.g., sugar composition) in different cultivated groups.

Another notable pathway was the terpenoid and polyketide-metabolism pathway, as this pathway was shared by three migration events (from the ancestor of NWC_W, NWC_C, and NC_C to KU_C, from XJ_C to KU_C, and from NC_C to West_C). The enriched subpathways of this pathway were different for the three events, where the first event primarily included diterpenoid biosynthesis, the second included zeatin biosynthesis, and the third included diterpenoid biosynthesis. These compounds have been reported to be components of apricot aroma and skin color^25^, and different compounds could give rise to different aroma characteristics and skin colors. Hence, the introgression of diverse subpathways was expected to lead to diverse aromatic characteristics and skin colors. Collectively, we concluded that introgression primarily mediated the high germplasm diversity by introducing various phenotype-related genes for different groups.

## Conclusions

In the present study, we demonstrated that the germplasms of Chinese apricot exhibit much higher genetic diversity than those cultivated in Western countries. This higher germplasm diversity was driven by frequent introgression events. Our results emphasize that introgressed regions provide an essential reservoir of genetic resources for modern crop-breeding sustainability and improvement.

## Materials and methods

### Plant materials and DNA extraction

Samples of *P. armeniaca* (“Yinxiangbai”) were planted at the Chinese National Germplasm Repository for Plums and Apricots located at Liaoning Institute of Pomology (Liaoning, China) for breeding and research purposes. A total of 180 apricot accessions were resequenced (Supplementary Table 8). Leaves from all accessions were sampled from the Chinese National Germplasm Repository for Plums and Apricots located at the Liaoning Institute of Pomology (Liaoning, China).

### DNA extraction, library construction, and sequencing

For each sample used for whole-genome sequencing and assembly, total genomic DNA was extracted from fresh young leaves after blooming using a Plant DNA Kit (TIANGEN, Beijing) according to the manufacturer’s instructions. The extracted DNA was then used for the construction of long-read and short-insert-size (350-bp) paired-end (PE) libraries.

For the long-read library, the genomic DNA was sheared to ~20 kb, followed by library construction and subsequent sequencing on the Oxford Nanopore platform promethION following the standard ONT protocol of the sequencing kit. To obtain clean ONT reads, adapter sequences and low-quality bases (quality score less than 7) at the beginning or end of the raw reads were trimmed, and short reads (less than 7 kb) were filtered using *Porechop* (version 0.2.4) (https://github.com/rrwick/Porechop).

For short-insert-size libraries, ~1 μg of DNA was sheared to 300–500 bp using a Covaris S2 Focused Ultrasonicator (Covaris), and the library was constructed and sequenced following the instructions for the BGISEQ-500 (BGI, China). Raw reads containing adapter sequences and those with ambiguous or low-quality bases at the beginning or end were trimmed using *SOAPnuke*^26^ (filter -l 16 -q 0.2 -n 0.05).

We further constructed a Hi-C library for this individual. To achieve this, DNA in the form of chromatin was fixed, digested (restriction enzymes GATC [MboI, DpnII], GAATTC [EcoRI]), ligated, and treated following the available protocol^27^. DNA was then sheared to a mean fragment size of 250 bp, and libraries were generated using NEBNext, Ultra enzymes, and Illumina-compatible adapters. The libraries were sequenced on an Illumina HiSeq X-Ten platform. Low-quality reads were filtered using *SOAPnuke*^26^ with the following parameters: “filter -l 16 -q 0.2 -n 0.05”. Clean Hi-C reads were evaluated using *HiC-Pro* (version 2.8.0)^28^.

To aid with annotation of the genome, we also generated RNA-seq data from different tissues. Total RNA was extracted from the young leaves, mature leaves, phloem tissues from branches, and mesocarp tissues from young fruit and mature fruit stages using Gambino’s method^29^. RNA libraries were constructed according to the standard mRNA protocol for the BGI kit. The libraries were sequenced using massive parallel sequencing by synthesis on a BGISEQ-500 in PE 150-bp mode. The obtained raw reads were filtered by removing adapter sequences and low-quality bases using *SOAPnuke*^26^ with the parameter “filter -l 16 -q 0.2 -n 0.05”.

For resequenced samples, genomic DNA was extracted from fresh young leaves using the modified cetyl trimethylammonium bromide (CTAB) method. Approximately 1 μg of genomic DNA from each accession was sheared to 300~500 bp using a Covaris S2 Focused Ultrasonicator (Covaris). A whole-genome shotgun paired-end library was prepared and sequenced using a BGISEQ-500 sequencer (BGI, China). The adapter sequences and the ambiguous and low-quality bases at the beginning or end of raw reads were trimmed using *SOAPnuke*^26^. All of the clean sequencing data were deposited at NCBI under BioProject number PRJNA577047.

### Genome-size estimation

The *K-*mer frequency distribution was used to estimate the genome size of “Yinxiangbai”. All clean short reads were used for 17-mer frequency-distribution analysis using *Jellyfish*^30^. To verify the *K*-mer result, we also estimated the genome size of *P. armeniaca* (“Yinxiangbai”) by flow cytometry. Approximately 20–50 mg of chopped fresh tissue was cultured in 1 mL of LB01 buffer, and the cells were collected and filtered through a cell strainer^31^. The samples were stained with 50 μg/mL PI and simultaneously digested with 50 μg/mL RNase in an ice bath for 30 min prior to analysis using a MoFlo-XDP flow cytometer (Beckman). Three biological repeats were performed, and 500 nuclei were measured for each replicate. The genome size of the apricot was estimated using the rice genome (*Oryza sativa* L. spp. *Japonica* var. *Nipponbare*, 380.2 Mb) as a control.

### Genome assembly

We adopted a *de novo* whole-genome assembly strategy that combined short PE reads and ONT long-read data. First, clean ONT reads were error-corrected using *Canu* (version 1.7)^32^ and then assembled using *SMARTdenovo* (version 1.0) (http://github.com/ruanjue/smartdenovo) under the optimized parameters. Next, the draft-assembled sequences were subjected to base correction using *Racon*^33^ (version 1.2.1, https://github.com/isovic/racon) with the parameter “racon <clean.fa> <overlaps.paf> <genome.fa>”. Finally, the genome was further polished with *Pilon*^34^ (version 1.22) using high-coverage (155×) BGISEQ-500 paired-end data.

### Pseudochromosome construction

Sequencing data from the Hi-C library were used to generate a chromosomal-level assembly of the genome. Briefly, we used *Bowtie2*^35^ to align the clean Hi-C paired-end reads to the assembled contigs, with low-mapping-quality reads being excluded to build raw inter-/intrachromosomal contact maps. Valid Hi-C reads with uniquely mapped read pairs were retrieved to count the number of valid interaction pairs and invalid interaction pairs. The open-source tools *Juicer* (version 1.5)^36^ and *3D-DNA* (version 180922)^37^ were used to cluster, order, and orient the *P. armeniaca* “Yinxiangbai” assembly to pseudochromosomes.

### Genome annotation

Known repeats in the *P. armeniaca* “Yinxiangbai” genome were identified using *RepeatMasker* (version 4.0.5)^38^ and *RepeatProteinMasker* (version 4.0.5) with *Repbase* (version 21.01) as the database^39^. *De novo* repeats were identified using *RepeatModeler* (version 1.0.5), *LTR_FINDER* (version 1.0.5), *PILER*, and *RepeatScout* followed by *RepeatMasker*.

Protein-coding gene predictions were performed using a combination of homology-based prediction, *de novo* prediction, and transcriptome-based methods. For homolog-based prediction, protein sequences from *P. persica*^13^, *P. mume*^14^, *P. avium*^15^, *P. dulcis*^40^, *P. yedoensis*^41^, *M. domestica*^42^, and *Arabidopsis thaliana*^43^ were used as training data sets. Protein sequences were mapped against the genome using *tBLASTn* implemented in the *BLAST* package (version 2.2.26), and the best hits for each homologous gene with the lowest *E*-value and the highest coverage were retained for further analysis. *De novo* prediction was performed based on the repeat-masked genome using *Augustus*, ab initio prediction software (version 3.2.2), and *SNAP*.

For transcriptome-based prediction, RNA-seq data generated from multiple tissues (young leaves, mature leaves, phloem tissues of branches, and mesocarp) were used for gene annotation using the *HiSat2* and *StringTie* pipelines^44–46^. Briefly, high-quality reads from RNA-seq were mapped to the genome using *HiSat2*. Subsequently, the transcripts were reconstructed using *StringTie*.

EVidenceModeler (version 1.1.1)^47^ was used to integrate all gene models from the three methods described above into a nonredundant gene set.

To conduct functional annotation of the predicted genes, the protein sequences were aligned against the NCBI-NR, euKaryotic Orthologous Groups (KOG), Swiss-Prot, and TrEMBL databases. To detect the protein domain, we performed Gene Ontology annotation by mapping protein sequences to the InterPro and Pfam databases using *InterProScan* and *HMME*^48^. Potential pathways were identified by mapping the protein sequences against the Kyoto Encyclopedia of Genes and Genomes (KEGG) database.

### Sequence alignment, variation calling, and annotation

The qualified reads from each resequenced individual were aligned to the assembled reference genome using Burrows–Wheeler Aligner (0.7.17-r1188)^49^ with the parameters “-k 19 -M -T 30”. Primary variation calling was conducted using the Genome Analysis ToolKit (GATK, v4.1.2)^50^. The filtering of low-quality variants using the criteria “QUAL <100 || DP <540 || DP> 5400 || MQ <55.0 || QD <2.0 || FS> 50.0 || BaseQRankSum <−3.0 || ReadPosRankSum <−2.0 || MQRankSum <−12.5 || SOR> 5.0” generated the final variant set. The variants located in gene regions were further annotated using Reseqtools (https://github.com/BGI-shenzhen/Reseqtools).

### Population structure and phylogenetic analysis

To minimize the bias caused by linkage disequilibrium (LD), only the variants where the distance of any two variants was >1 kb were used to construct the population structure. The population structure was inferred using ADMIXTURE (v1.3)^51^ by setting the predefined genetic cluster number *K* from two to seven. To evaluate the best genetic cluster number *K*, the cross-validation (CV) error was tested for each *K*, and the lowest CV error was regarded as the best.

A distance matrix for all apricot samples was calculated based on the final variant set using VCF2Dis (https://github.com/BGI-shenzhen/VCF2Dis). The calculated distance matrix was then subjected to unrooted phylogenetic neighbor-joining tree construction using PHYLIP v3.68 (http://evolution.genetics.washington.edu/phylip.html).

### Genetic diversity calculation

The nucleotide diversity (*π*)^52^, heterozygosity (*H*_*E*_)^53^ for each apricot group, and fixation index *F*_*ST*_^54^ between group pairs were estimated based on their definitions in a window size of 20 kb with a step size of 10 kb using the in-house Perl scripts from a previous report^54^. Only windows comprising ≥8,000 effective sites were considered.

### Introgression

Patterson’s *D*-statistics in a four-population mode (P1, P2, and P3 outgroup), where P1, P2, and P3 represent any apricot groups of interest, were calculated using *qpDstat* implemented in ADMIXtools (V5.1)^55^ to test introgressions among apricot groups based on the use of peach (*P. persica*) as the outgroup. A negative *D* value indicated that P2 shares more derived alleles with P3 than does P1, thus indicating introgression occurrence between P2 and P3. The significance was assessed using a block jackknife procedure with a block size of 5 Mb. To identify the introgression region, sliding-window (window size of 20 kb and step size of 10 kb) analyses of a modified *f*-statistic (*f*_*d*_) were performed using ABBABABAwindows.py (https://github.com/simonhmartin/genomics_general)^56^. The output windows with SNP numbers <30 were excluded, and the windows with the top 5% *f*_*d*_ values were considered candidate introgression regions according to a previous report^57^. The *f*_*d*_ values and the introgression regions were displayed along eight chromosomes using RectChr v1.24 (https://github.com/BGI-shenzhen/RectChr).

TreeMix22 (v1.12)^58^ was used to model the gene flow between the apricots. This method first infers a maximum likelihood tree based on genome-wide allele-frequency data and then identifies potential gene flow from a residual covariance matrix. Admixture scenarios possessing *m* = 1 to *m* = 7 migration events were modeled.

### Functional enrichment

To further understand the potential functions of the genes in these candidate-selection or introgression regions, gene enrichment was performed using the *phyper* function within the R platform based on the Gene Ontology (GO) and KEGG pathway annotation from the “Yinxiangbai” genome, and FDR corrections (GO enrichment) or *q*-values (KEGG pathway enrichment) were applied by multiple testing^59^. GO terms and KEGG pathways exhibiting an FDR or *q*-value <0.05 were considered to be significantly enriched, while those possessing corrected *P-*values <0.05 (Fisher’s exact test)^57^ were also reported in the supporting information.

## Supplementary information

Supplementary Table

Supplementary Information

## Data Availability

The *P. armeniaca* “Yinxiangbai” genome project was deposited into the NCBI under BioProject PRJNA577047 and BioSample SAMN13017833. The assembled genome and annotation are available at CNSA and GenBank. Raw read files from the genome sequencing are available at the NCBI Sequence Read Archive (SRR10322053, SRR10322054, and SRR10322055) and CNSA (CNP0000718). The version described in this paper is version paYXB01000001. All the RNA-seq data (SRR10339150, SRR10339151, SRR10339152, SRR10339153, and SRR10339154) and functional annotation files of the *P. armeniaca* “Yinxiangbai” genome are available at the CNSA.
