# Supplementary material for: Frequent germplasm exchanges drive the high genetic diversity of Chinese-cultivated common apricot germplasm
Source: Hortic Res. 2021 Oct 1;8:215. doi: 10.1038/s41438-021-00650-8 (PMC8484454; doi:10.1038/s41438-021-00650-8)
Supplement: Supplementary file 2 — Supplementary Information [file 41438_2021_650_MOESM2_ESM.docx]

**Supplementary Information**

**Frequent germplasm exchanges drive the high genetic diversity of Chinese cultivated common apricot germplasm**

Qiuping Zhang^1^, Diyang Zhang^2^, Kang Yu^3^, Jingjing Ji^4^, Ning Liu^1^, Yuping Zhang^1^, Ming Xu^1^, Yu-Jun Zhang^1^, Xiaoxue Ma^1^, Shuo Liu^1^, Wei-Hong Sun^2^, Xia Yu^2^, Wenqi Hu^2^, Si-Ren Lan^2^, Chi Zhang^4^, Zhong-Jian Liu^2,5,6†^, Weisheng Liu^1†^

**Affiliations:**

1. Liaoning Institute of Pomology, Yingkou 115009, China

2. Key Laboratory of National Forestry and Grassland Administration for Orchid Conservation and Utilization at College of Landscape Architecture, Fujian Agriculture and Forestry University, Fuzhou 350002, China

3. BGI Institute of Applied Agriculture, BGI-Agro, Shenzhen 518210, China

4. BGI Genomics, BGI-Shenzhen, Shenzhen 518083, China

5. Institute of Vegetable and Flowers, Shandong Academy of Agricultural Sciences, Jinan 250100, China

6. Zhejiang Institute of Subtropical Crops, Zhejiang Academy of Agricultural Sciences, Wenzhou 325005, China

Running title: Apricot genome and population genetic diversity

**Content**

[Supplementary Figures 4](#_Toc72944018)

[Supplementary Figure 1 Genome size and heterozygosity estimation using 17 *K-mer* distribution. 4](#_Toc72944019)

[Supplementary Figure 2 Linkage map of the assembled ‘Yinxiangbai’ genome. 5](#_Toc72944020)

[Supplementary Figure 3 Intensity signal heat map of Hi-C chromosome. 6](#_Toc72944021)

[Supplementary Figure 4 Cross-validation (CV) errors of ADMIXTURE runs. 7](#_Toc72944022)

[Supplementary Tables 8](#_Toc72944023)

[Supplementary Table 1 The statistics of raw data using PacBio and Nanopore sequencing. 8](#_Toc72944024)

[Supplementary Table 2 Assembly statistics of the *P. armeniaca* ‘Yinxiangbai’ genome. 9](#_Toc72944025)

[Supplementary Table 3 The length of chromosome by Hi-C assembly of *P. armeniaca* ‘Yinxiangbai’ genome. 10](#_Toc72944026)

[Supplementary Table 4 BUSCO assessment of gene annotation of *P. armeniaca* ‘Yinxiangbai’ genome. 11](#_Toc72944027)

[Supplementary Table 5 The prediction of gene structures of *P. armeniaca* ‘Yinxiangbai’. 12](#_Toc72944028)

[Supplementary Table 6 The statistics results of function annotation. 13](#_Toc72944029)

[Supplementary Table 7 The statistic results of repeat sequence in *P. armeniaca* ‘Yinxiangbai’ genome. 14](#_Toc72944030)

[Supplementary Table 8 Sample information of apricots sequenced in this study. (see separate files) 15](#_Toc72944031)

[Supplementary Table 9 Mapping statistics of the resequencing data for the 180 apricot accessions against the reference genome. (see separate files) 15](#_Toc72944032)

[Supplementary Table 10 The statistics of SNP and InDel identified in 180 apricot accessions. (see separate files) 15](#_Toc72944033)

[Supplementary Table 11 Genetic variation among or within apricot groups. (see separate files) 15](#_Toc72944034)

[Supplementary Table 12 *D* statistics for different four-taxon (P1, P2, P3, Peach) of apricot groups. (see separate files) 15](#_Toc72944035)

[Supplementary Table 13 The statistics of the candidate introgressed regions for different four-taxon (P1, P2, P3, Peach). (see separate files) 16](#_Toc72944036)

[Supplementary Table 14 Gene ontology (GO) enrichment of genes in introgressed regions for migration events (m = 1 to 7). (see separate files) 17](#_Toc72944037)

[Supplementary Table 15 KEGG pathway enrichment of genes in introgressed regions for migration events (m = 1 to 7). (see separate files) 17](#_Toc72944038)

# Supplementary Figures


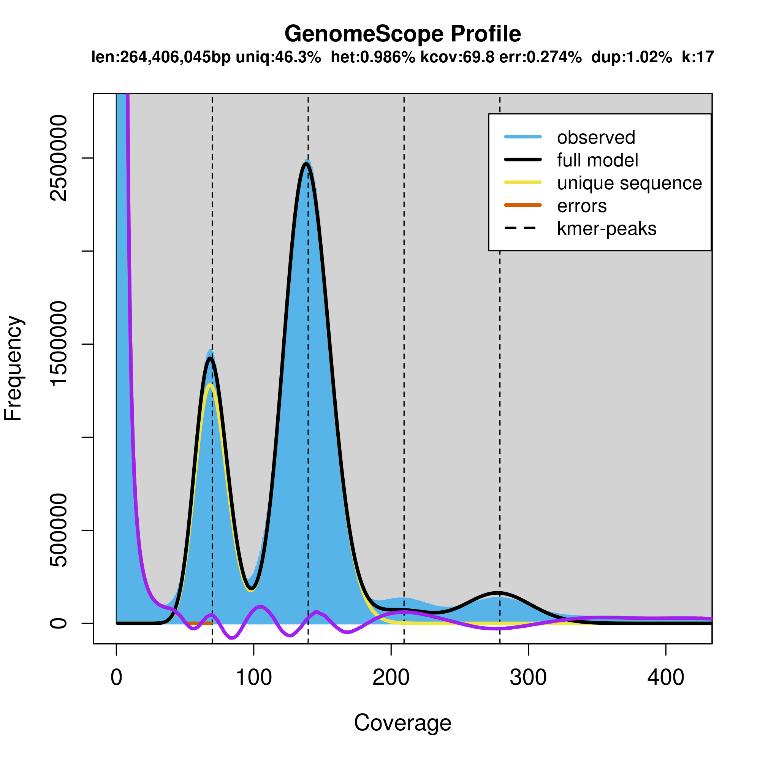


## Supplementary Figure 1 Genome size and heterozygosity estimation using 17 *K-mer* distribution.


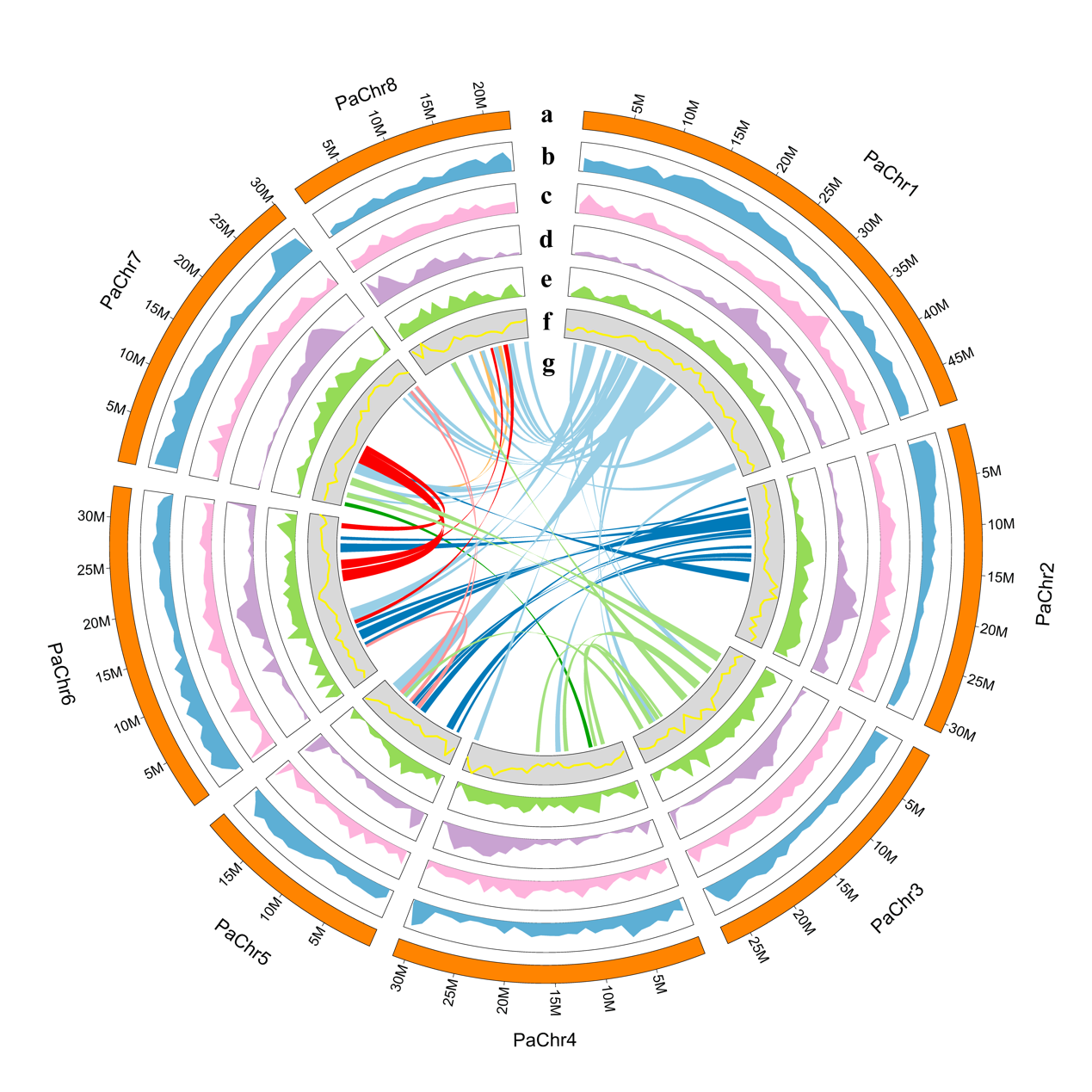


## Supplementary Figure 2 Linkage map of the assembled ‘Yinxiangbai’ genome. a. Chromosomes of *P. armeniaca* ‘Yinxiangbai’ PaChr1-PaChr8. b. Gene number. c. DNA transposable elements. d. Gypsy-type transposable elements (TEs). e. Copia-type TEs. f. GC contents. g. Self-syntenic genome regions.

**
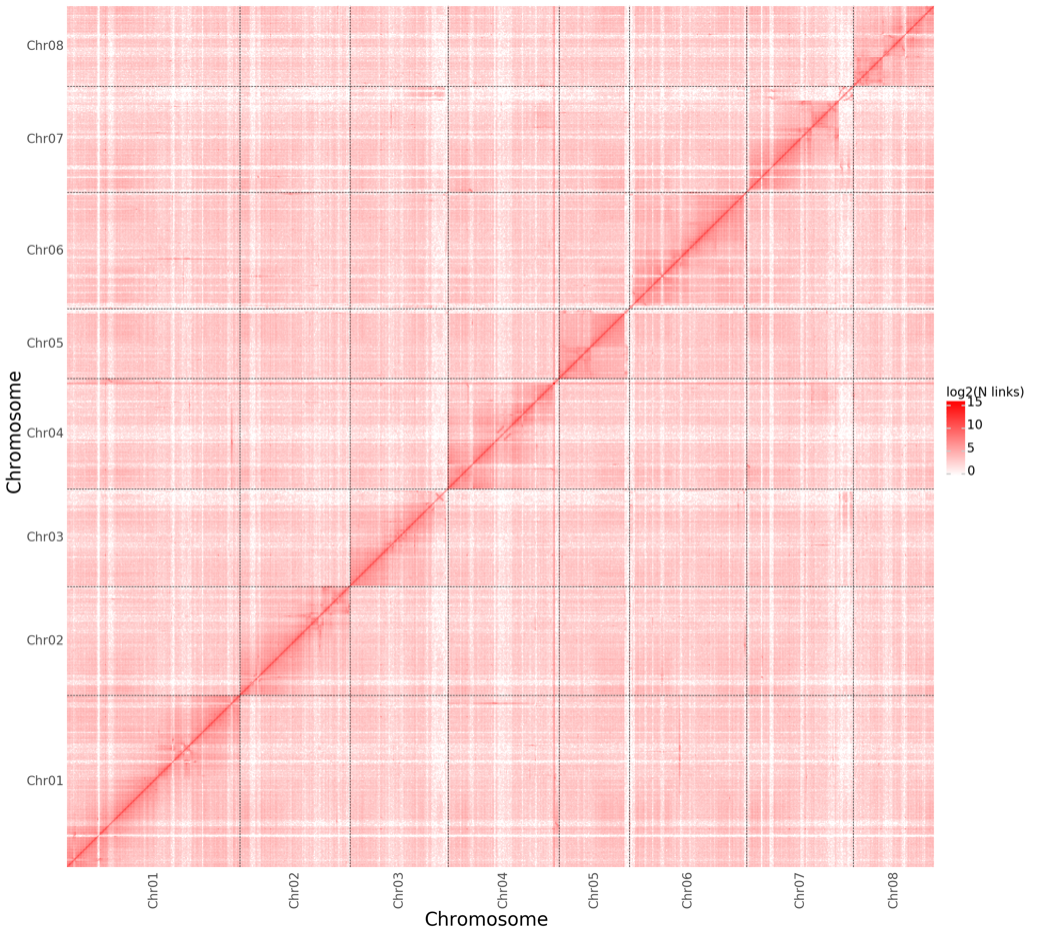
**

## Supplementary Figure 3 Intensity signal heat map of Hi-C chromosome.


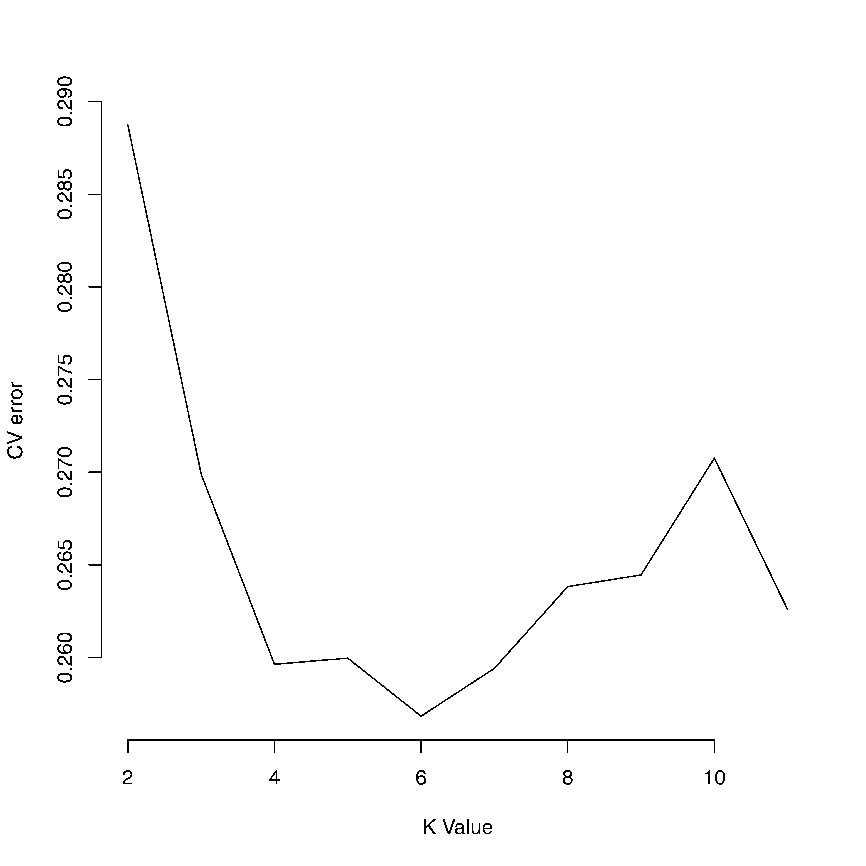


## Supplementary Figure 4 Cross-validation (CV) errors of ADMIXTURE runs.

# Supplementary Tables

## Supplementary Table 1 The statistics of raw data using PacBio and Nanopore sequencing.

| **PacBio** | **ID** | **ZMWNUM** | **Total bases** | **Total reads** | **Average** | **Max** | **N50** |
| --- | --- | --- | --- | --- | --- | --- | --- |
|  |  |  | **(Gb)** |  | **length** | **length** | **length** |
|  | Yinxiangbai | 5,920,168 | 47.52 | 6,159,163 | 7,715.83 | 193,246 | 14,150 |
| **Nanopore** | **Sample** | **Total Reads** | **Total Base (GB)** | **Max** | **Mean** | **N50 Length (bp)** | **MeanQV** |
|  |  |  |  | **Length(bp)** | **Length(bp)** |  |  |
|  |  |  |  |  |  |  |  |
|  | Yinxiangbai | 7,300,764 | 82.37 | 117,459 | 11,282 | 11,752 | 9.1 |

## Supplementary Table 2 Assembly statistics of the *P. armeniaca* ‘Yinxiangbai’ genome.

|  | **Contig** | |
| --- | --- | --- |
|  | **Length(bp)** | **Number** |
| Max length | 15,297,900 |  |
| N50 | 4,038,258 | 17 |
| N60 | 3,487,594 | 24 |
| N70 | 2,572,382 | 33 |
| N80 | 1,881,471 | 44 |
| N90 | 988,841 | 63 |
| Total length | 251,190,293 |  |
| number>=1000bp | 256 |  |
| number>=10000bp | 239 |  |
| GC rate （%） | 37.55 |  |

## Supplementary Table 3 The length of chromosome by Hi-C assembly of *P. armeniaca* ‘Yinxiangbai’ genome.

|  | **Length** | **No. of contigs** |
| --- | --- | --- |
| Chr01 | 48,757,601 | 26 |
| Chr02 | 30,979,433 | 34 |
| Chr03 | 27,445,780 | 48 |
| Chr04 | 31,253,906 | 32 |
| Chr05 | 19,750,602 | 32 |
| Chr06 | 33,056,764 | 49 |
| Chr07 | 30,082,201 | 41 |
| Chr08 | 22,582,249 | 25 |
| Contig N50 Length (bp) | | 3,166,142 |
| Scaffold N90 Length (bp) | | 19,750,602 |
| Scaffold N50 Length (bp) | | 30,979,433 |
| Total Size (bp) | | 251,329,793 |
| Total length of contigs (bp) | | 251,190,293 |
| Length of unanchored contigs (bp) | | 7,421,257 |
| Anchored contig rate (%) | | 97.04 |

## Supplementary Table 4 BUSCO assessment of gene annotation of *P. armeniaca* ‘Yinxiangbai’ genome.

| **Type** | **Number** | **Percentage（%）** |
| --- | --- | --- |
| Complete BUSCOs (C) | 1,323 | 96.2 |
| Complete and single-copy BUSCOs (S) | 1,250 | 90.9 |
| Complete and duplicated BUSCOs (D) | 73 | 5.3 |
| Fragmented BUSCOs (F) | 7 | 0.5 |
| Missing BUSCOs (M) | 45 | 3.3 |
| Total BUSCO groups searched | 1,375 |  |

## Supplementary Table 5 The prediction of gene structures of *P. armeniaca* ‘Yinxiangbai’.

|  | **Gene set** | **Number** | **Average** | **Average** | **Average** | **Average** | **Average** |
| --- | --- | --- | --- | --- | --- | --- | --- |
|  |  |  | **Gene** | **CDS** | **exon per** | **exon** | **intron** |
|  |  |  | **lenth(bp)** | **length(bp)** | **gene** | **length(bp)** | **length(bp)** |
| ***De novo*** | ***AUGSTUTUS*** | 22973 | 2610.13 | 1188.7 | 5.06 | 235.11 | 350.46 |
|  | ***GENSCAN*** | 19230 | 7817.69 | 1441.71 | 6.65 | 216.72 | 1128.02 |
|  | ***GlimmerHMM*** | 36355 | 4118.72 | 819.91 | 3 | 273.46 | 1650.86 |
|  | ***SNAP*** | 31554 | 2440.87 | 662.85 | 3.79 | 174.78 | 636.71 |
| **Homolog** | ***A. thaliana*** | 23629 | 2087.09 | 933.53 | 4.2 | 222.1 | 360.13 |
|  | ***M. domestica*** | 23593 | 2889.69 | 1044.87 | 4.82 | 216.59 | 482.41 |
|  | ***P. mume*** | 31998 | 2008.37 | 854.34 | 4.07 | 209.92 | 375.93 |
|  | ***P. dulcis*** | 30152 | 2095.29 | 917.16 | 4.29 | 213.78 | 358.08 |
|  | ***P. avium*** | 33727 | 1896.18 | 780.61 | 3.78 | 206.68 | 401.72 |
|  | ***P. persica*** | 31041 | 2017.35 | 898.91 | 4.23 | 212.69 | 346.66 |
|  | ***P. yedoensis*** | 30619 | 1919.21 | 827.86 | 4.09 | 202.56 | 353.54 |
|  | ***TF*** | 5799 | 1794.71 | 743.61 | 2.54 | 292.57 | 681.81 |
| **RNAseq** | | 23623 | 4496.57 | 3368.59 | 11.26 | 299.08 | 85.54 |
| **Integration** | **EVM** | 29230 | 2791.08 | 2165.42 | 9.28 | 233.3 | 52.57 |

## Supplementary Table 6 The statistics results of function annotation.

| **Annotation DataBase** | **Number** | **Percentage (%)** |
| --- | --- | --- |
| **Total** | 29,230 | 100% |
| **Nr** | 26,195 | 89.62 |
| **InterPro** | 23,808 | 81.45 |
| **GO** | 15,403 | 52.7 |
| **KEGG** | 19,566 | 66.94 |
| **Swiss-Prot** | 20,042 | 68.57 |
| **TrEMBL** | 26,501 | 90.66 |
| **Annotated** | **26743** | **91.49** |

## Supplementary Table 7 The statistic results of repeat sequence in *P. armeniaca* ‘Yinxiangbai’ genome.

| **Type** | **Repeat Size (bp)** | **% of genome** |
| --- | --- | --- |
| **Trf** | 11,311,720 | 4.500748 |
| **Proteinmask** | 15,484,082 | 6.160862 |
| **RepeatMasker (*De novo*, Known)** | 114,630,093 | 45.609433 |
| **Total** | 117,560,004 | 46.775196 |

## Supplementary Table 8 Sample information of apricots sequenced in this study (see separate files).

## Supplementary Table 9 Mapping statistics of the resequencing data for the 180 apricot accessions against the reference genome (see separate files).

## Supplementary Table 10 The statistics of SNP and InDel identified in 180 apricot accessions (see separate files).

## Supplementary Table 11 Genetic variation among or within apricot groups (see separate files).

## Supplementary Table 12 *D* statistics for different four-taxon (P1, P2, P3, Peach) of apricot groups (see separate files).

## Supplementary Table 13 The statistics of the candidate introgressed regions for different four-taxon (P1, P2, P3, Peach). (see separate files)

| **Migration** | **Four-taxon (P1, P2, P3, Peach)** | **Length(bp)** | **Proportion (%)** | **Gene Numbers** |
| --- | --- | --- | --- | --- |
| m1 | NC_C, NWC_W, XJ_C, Peach | 7,789,758 | 3.10 | 1033 |
|  | NEC_C, NWC_W, XJ_C, Peach | 10,389,700 | 4.13 | 1412 |
|  | KU_C, NWC_W, XJ_C, Peach | 10,089,703 | 4.01 | 1430 |
|  | PsPma_W, NWC_W, XJ_C, Peach | 11,749,679 | 4.67 | 1647 |
|  | Union | 21,769,427 | 8.66 | 2957 |
| m2 | PsPma_W, KU_C, NWC_C, Peach | 10,829,697 | 4.31 | 1297 |
|  | PsPma_W, KU_C, NC_C, Peach | 10,659,688 | 4.24 | 1213 |
|  | PsPma_W, KU_C, NWC_W, Peach | 10,629,693 | 4.23 | 1343 |
|  | Union | 19,399,478 | 7.72 | 2340 |
| m3 | PsPma_W, NEC_C, NC_C, Peach | 10,769,714 | 4.28 | 1404 |
| m4 | NC_C, NWC_C, XJ_C, Peach | 7,399,742 | 2.94 | 877 |
|  | NEC_C, NWC_C, XJ_C, Peach | 10,199,743 | 4.06 | 1437 |
|  | KU_C, NWC_C, XJ_C, Peach | 9,419,728 | 3.75 | 1286 |
|  | PsPma_W, NWC_C, XJ_C, Peach | 11,459,688 | 4.56 | 1541 |
|  | Union | 21,939,402 | 8.73 | 2889 |
| m5 | KU_C, West_C, NC_C, Peach | 8,469,788 | 3.37 | 1145 |
|  | XJ_C, West_C, NC_C, Peach | 7,999,804 | 3.18 | 1057 |
|  | XJ_W, West_C, NC_C, Peach | 8,189,814 | 3.26 | 1111 |
|  | PsPma_W, West_C, NC_C, Peach | 10,299,768 | 4.10 | 1416 |
|  | Union | 17,969,589 | 7.15 | 2354 |
| m6 | PsPma_W,KU_C, XJ_C, Peach | 10,249,710 | 4.08 | 1245 |
| m7 | PsPma_W,NEC_C, West_C, Peach | 9,469,761 | 3.77 | 1165 |
| Union m1~7 | Union | 69718456 | 27.73 | 8980 |

## Supplementary Table 14 Gene ontology (GO) enrichment of genes in introgressed regions for migration events (m = 1 to 7). (see separate files)

## Supplementary Table 15 KEGG pathway enrichment of genes in introgressed regions for migration events (m = 1 to 7). (see separate files)
